# Contribution of EEG in analyzing the intraregional « disconnectivity » in schizophrenia

**DOI:** 10.1192/j.eurpsy.2025.1702

**Published:** 2025-08-26

**Authors:** L. Triki, N. Smaoui, D. Jardak, R. Feki, I. Gassara, M. Bou Ali Maalej, N. Charfi, J. Ben Thabet, M. Maalej, S. Omri, L. Zouari

**Affiliations:** 1 Functional Explorations, Habib Bourguiba University Hospital; 2 psychiarty C, Hedi Chaker University Hospital, Sfax, Tunisia

## Abstract

**Introduction:**

« Disconnectivity » in schizophrenia seems to be subsequent to abnormalities of the white matter and distorted functional connectivity. That would explain the cognitive impairment, including the slowing of information processing, observed in these patients that could be one of the risk factors for conversion into clinical psychosis and a significant predictor of functional outcome.

**Objectives:**

This study aimed to search for signs of « disconnectivity » in quantitative electroencephalograms (EEG) in schizophrenic patients compared to healthy controls.

**Methods:**

It was a case-controlled study involving 15 schizophrenic patients and 15 healthy controls. The study was carried out at units of Psychiatry Department “C” and Functional Explorations Department Sfax’ hospital in Tunisia. Participants underwent a standard wakefulness EEG recording with a resting state period and a mental calculation test.The spectral density analysis for each frequency band was studied. The absolute spectral densities (ASD) of the following frequency bands were analyzed at resting state and after calculation moment: delta [0,5 – 3,5 Hz], thêta [4 – 7,5 Hz] alpha 1 [8 – 10 Hz] alpha 2 [10,5 – 12,5 Hz] and bêta 1 [13 – 20 Hz] in bilateral frontal and occipital regions. The Pearson’s correlation was applied between the two moments in the same region (for the same electrode). Good connectivity was found if p <0,05, the inverse meant « disconnectivity ».

**Results:**

Good connectivity was found for schizophrenics in the frontal region ( for alpha1 band in the right one (r=0,721 ; p=0,002) and in the left one (r=0,597 ; p=0,019), for beta1 band in the left one (r=0,616 ; p=0,014), and for thêta band in the right one (r= 0,569 ; p=0,027) and the left one (r=0,661 ; p=0,007)) and in the occipital region (for alpha2 band in the right one (r=0,726 ; p=0,002), for bêta1 in the right one (r=0,565 ; p=0,028), and for thêta band in the right one (r=0,836 ; p<0,001) and in the left one (r=0,829 ; p<0,001)). Disconnectivity was admitted for other bands in the same regions. However,for healthy controls, highly significant correlations (p<0.001) were observed in the right and left frontal and occipital regions for all frequency bands.

**Conclusions:**

These results support the « disconnectivity » theory in which it seems that relay neurons connecting the nerve cells between the thalamus and the cortex could not control the thalamic neurons. A dysfunction of these relay neurons, mainly GABAergic, is known as one of the main etiopathogenesis of schizophrenia.

**Disclosure of Interest:**

None Declared

